# Integrating traditional ecological knowledge into habitat restoration: implications for meeting forest restoration challenges

**DOI:** 10.1186/s13002-023-00606-3

**Published:** 2023-08-10

**Authors:** Shiekh Marifatul Haq, Andrea Pieroni, Rainer W. Bussmann, Ahmed M. Abd-ElGawad, Hosam O. El-Ansary

**Affiliations:** 1 Department of Ethnobotany, Institute of Botany, Ilia State University, Tbilisi, Georgia; 2 Wildlife Trust of India, Noida, Uttar Pradesh 201301 India; 3 University of Gastronomic Sciences of Pollenzo, Piazza V. Emanuele II 9, 12042 Pollenzo, Bra, Italy; 4 Department of Botany, Institute of Life Sciences, State Museum of Natural History, Karlsruhe, Germany; 5 Department of Plant Production, College of Food and Agriculture Sciences, King Saud University, P.O. Box 2460, 11451 Riyadh, Saudi Arabia

**Keywords:** Ethnobotanical uses, Elephant corridor, Biodiversity, Pioneer species, Habitat restoration

## Abstract

**Background:**

Traditional ecological knowledge (TEK) helps tribal communities adapt to socio-ecological changes, improving the long-term sustainability of their livelihood strategies and fostering social–ecological resilience. TEK provides thorough understanding of ecosystem dynamics, as well as how they relate to societal norms, practices, and resource use patterns. The integrity of TEK is often in jeopardy due to changes in belief systems, regional languages, traditional ways of subsistence, and disruption of traditional social–ecological systems. Landscape restoration has the ability to promote self-determination while safeguarding the livelihoods, beliefs, cultural, and biodiversity of indigenous peoples. However, there is a substantial knowledge gap on how TEK might aid ecosystem restoration, particularly in elephant corridors.

**Methods:**

The current study focused on gathering traditional ecological knowledge on the woody tree species from the Dering-Dibru Saikhowa Elephant Corridor using semi-structured interviews, group discussions, and direct observations. The acquired data were applied to heat map cluster analysis and ordination techniques using R software version 4.0.0.

**Results:**

Traditional usage information of 31 tree species utilized for food, fodder, timber, fuelwood, medicinal, and livelihood by local people was gathered. Most of the species utilized locally belonged to the families Combretaceae and Fabaceae. The species were classified into single, double, or multi-uses based on the extent of utilization. *Azadirachta indica, Phyllanthus emblica*, and *Syzygium cumini* (six each) had the highest utilization, while *Mesua ferrea* had the lowest. *Chionanthus ramiflorus, Artocarpus heterophyllus*, and *Dillenia indica* were among the plants valuable to wildlife, providing both forage and habitat for a wide variety of birds and animals. *Artocarpus heterophyllus, Averrhoa carambola, Mangifera indica, P. emblica, Psidium guajava,* and *S. cumini* were among the plants important for the livelihoods of the local community. Our findings demonstrated that local people were knowledgeable about the plant species to use as pioneer species, such as *Bombax ceiba, Albizia lebbeck, D*.* indica, S. cumini, P. emblica, Lagerstroemia speciosa*, and *Alstonia scholaris,* for habitat restoration in a diverse habitat. We classified the habitat of the enlisted species into different categories, and two clusters (clusters 1 and 2) were identified based on the similarity of woody species in different habitats. We prioritized multiple tree species for eco-restoration using the information collected through TEK. We planted 95,582 saplings on 150 hectares in the Dering-Dibru Saikhowa Elephant Corridors’ degraded habitat patches, which will serve as future reference site for landscape rehabilitation. Out of total saplings planted, 56% of the species were linked to native communities through ethnobotanical uses, as well as providing connectivity and habitat for elephant movement, 16% of all woody species are pioneer species to colonize a degraded habitat, 15% of all woody species are preferred food and foraging by wildlife, and 13% of the species as a source of livelihood for local people, incorporating social, economic, cultural, and biodiversity benefits into the restoration framework.

**Conclusion:**

The current study also provides insights how the TEK can assist with aspects of ecological restoration, from reference ecosystem reconstruction and adaptive management through species selection for restoration, monitoring, and evaluation of restoration effectiveness.

## Introduction

Indigenous and local communities’ territories include about 80% of the world’s remaining forest biodiversity, and the lands they manage release 73% less carbon than those managed by other groups [1, 2]. Therefore, despite current conventions that tie modern land managers to the scientific discovery, indigenous peoples’ lands have thrived resiliently. A mosaic of cultures, developing in related settings, has led to the development of a wide range of indigenous ecological knowledge and belief systems based on sustainability ideals [3]. As a result, TEK offers crucial ecological insights as well as a network of knowledge that incorporates principles that might aid in ecosystem restoration.

Traditional peoples all across the world are well aware of the natural resources on which they rely [4]. Such knowledge has been aided in the development of scientific management plans and is becoming more widely recognized as a source of data for natural resource conservation, management, and sustainable usage [5]. Traditional ecological knowledge (TEK) integration may aid adaptive management given that it frequently supplements previously gathered ecological data by providing additional information at a finer spatial scale than scientific data [6]. An in-depth understanding of ecosystems and their dynamics, as well as their linkages to societal values, activities, and resource use patterns, is necessary for ecological restoration [7].

Comprehensive restoration demands a multifaceted approach that takes historical, social, cultural, political, esthetic, and moral considerations into account [8]. Restoring biodiversity and functionality needs local community support, correct policies, appropriate legislation, long-term funding, and scientific and technological expertise [9]. Ecological restoration projects should be successful in restoring and sustaining ecological integrity, efficient in using practical and cost-effective means to achieve goals, and inclusive of cultural and natural interrelationships, which may be achieved by merging scientific ecological and traditional ecological knowledge [10, 11]. This study was conducted as part of the planning and establishment one of the most important elephant corridor projects, the D’ering-Dibru Saikowa Elephant Corridor. The corridor was planned in collaboration with the local community and State Forest Departments through “Connecting Landscapes, Empowering People, and Protecting Elephants” as part of Wildlife Trust of India’s Land Securement Strategy. This entailed designating critical corridor land as a “Community Conserved Area,” restoring degraded corridor habitats, sensitizing the local population and gaining its support for wildlife conservation, providing green livelihood options to local communities, building capacity and empowering management authorities, managing Human–Elephant Conflict situations, and deploying local community-based organizations as Green Corridor Champions. The goal was to establish a win–win situation for elephants, residents, and administrative officials.

Ecological restoration is gaining popularity, and it is becoming increasingly clear that cultural practices, as well as ecological processes, should be considered [12]. TEK may provide a robust foundation for ecological restoration because it co-evolved with ecosystems [13]. Unfortunately, combining indigenous knowledge systems into “top-down” ecological restoration efforts still often remains a significant problem [14]. Community engagement is critical during the restoration process, especially when working with communities that have a wealth of traditional knowledge related to biodiversity and natural resource management [15]. In remote areas, where traditional people’s contributions to ecosystem conservation and management have been recognized, cultural and social aspects of ecological restoration become even more important.

In recent decades, the potential contribution of TEK to natural resource conservation, management, and sustainable use has been more widely recognized, documented, and applied [16, 17]. Although in Northeast India (NEI), traditional ecological knowledge has long been documented, we still have a long way to go toward linking ecological processes with societal values using TEK systems as a key tool [18]. The inhabitants of NEI rely mostly on forest ecosystem services and agriculture for food and sustenance [19]. Shifting cultivation (locally known as Jhum) is a traditional agricultural strategy for preparing crop fields in hilly areas that involves cutting and burning natural plants. In shifting cultivation, the same land parcel is used for forestry and agriculture at different periods, combining forestry in agriculture and vice versa [20]. This combined approach must be considered the context of human-managed ecosystems, building on the traditional ecological knowledge base that under pins the ability to provide long-term natural resource management [21].

While TEK’s value in ecological restoration has more recently been recognized [2], its current or potential contribution has not been well examined in India. The TEK contribution to the maintenance and restoration of elephant corridors has not been documented, and to the best of our knowledge, no attempts have been made to use TEK as ecological restoration tool for animal corridors. In this study, we focused (1) on the reintroduction of woody vegetation that are useful (e.g., as food, timber, fodder, medicine, fuelwood, and livelihood) for the native communities; (2) providing habitat and forage for wildlife; (3) furthermore, we documented the associated ecology parameters of the woody vegetation (i.e., habitat types and indigenous knowledge in recovering plant cover after disturbance (pioneer species)); and (4) finally, we provided local communities with direct field-based training for future plantings in the landscape. By using the above factors, we prioritized multi-tree species for restoration of the degraded elephant corridors that can be selecting as reference sites for landscape restoration in the future. The study will help practitioners to quickly identify and evaluate species, their appropriateness, and important species interactions. Additionally, by enabling the use of cultural practices, TEK can aid in defining native reference ecosystems and stimulate restoration. Strategies for restoring degraded ecosystems that combine TEK and ecological principles may be very successful. The findings of this study will lay the groundwork for combining traditional knowledge into ecological restoration initiatives in additional elephant corridors around the area.

## Material and methods

### Study area

**Fig. 1:**
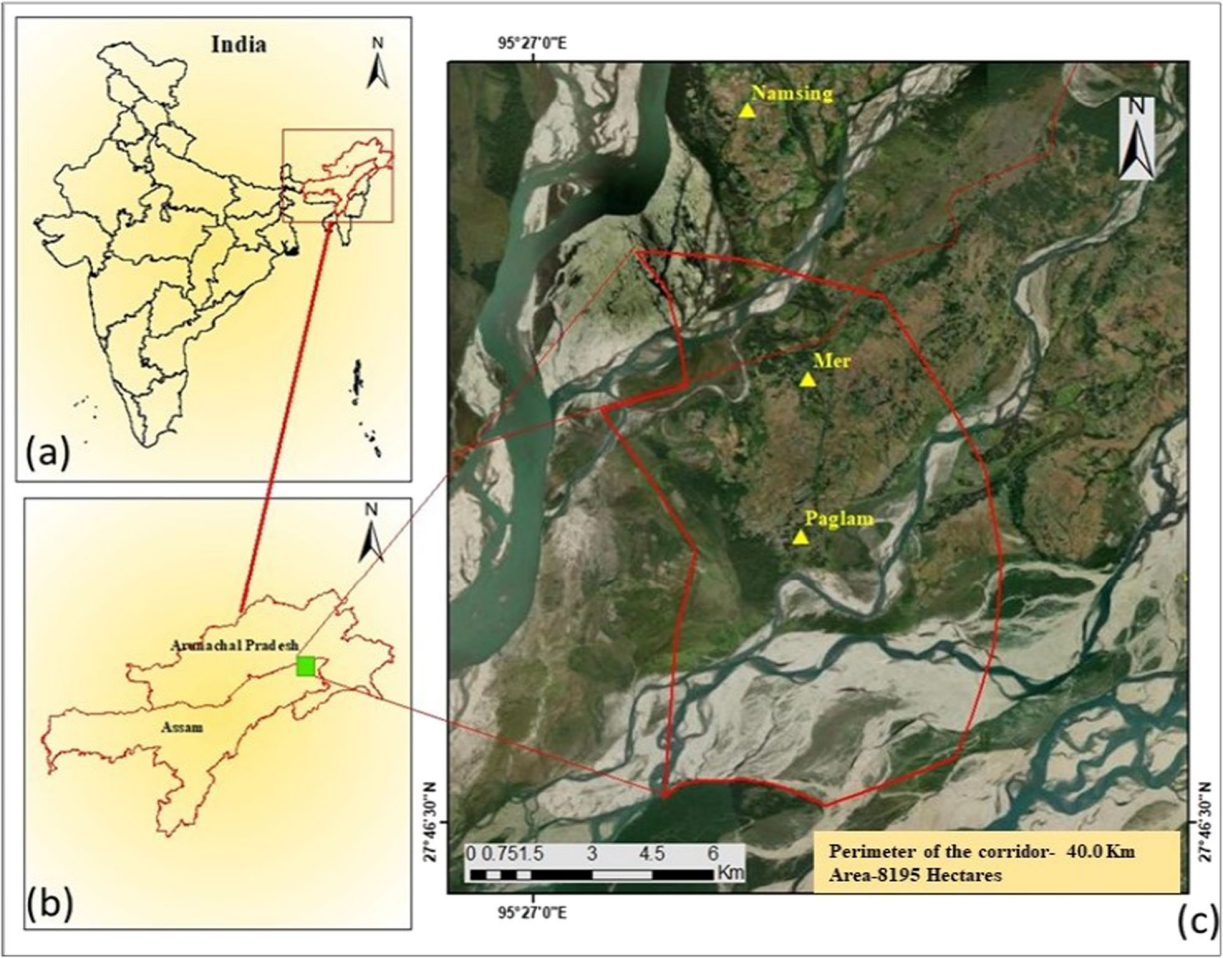
Map of the study area (**a**) India, (**b**) Arunachal Pradesh and Assam states, and (**c**) three surveyed villages in Dering-Dibru Saikhowa Elephant Corridor in Arunachal Pradesh, India

The study area elephant corridor links the Dibru Saikhowa National Park in Assam with the Daying Ering Wildlife Sanctuary in Arunachal Pradesh. The study was implemented in three villages (Mer, Palgam, and Namsing) Dibang Valley district of Arunachal Pradesh in India (Fig. 1). The Dibang Valley district includes two districts, i.e., Dibang Valley and Lower Dibang Valley. Before meeting the Brahmaputra, the Dibang River passes through Dibang Valley district. The valley is located between 27°30″N′ and 28°33′ latitude and 95°15″E′ and 96°30″E′ longitude. The district is bordered on the north by Dibang Valley district, on the east by Lohit District and the McMahon Line (China), on the west by East Siang district and Upper Siang district, and on the south by Tinsukia district’s Sadiya sub-division [22]. In terms of area, this is the state’s largest district. The tributaries of the Dibang River, especially the Dri, Mathuan, Ithun, Taloh, Emra, Ahin, and Sisiri rivers, have produced a variety of sub-valleys. The area is located in a rainy belt with annual rainfall ranging from 3000 to 5000 mm. Lower Dibang Valley’s agroclimatic zones are subtropical and subhumid.

Lower Dibang Valley district’s forest areas are largely in the mountainous region, extending down to the riverbanks. Lower Dibang Valley district’s forest areas are largely in the mountainous region, extending up to the riverbank. At least 75% of the area is covered by alluvial vegetation dominated by grasses, 15% by semi-evergreen forests, and 10% by watercourses [23]. Many of these forests are unclassified legally and managed by local communities. The principal occupants of these areas are the Mishing, Adi, and few Galos communities, who rely on the forest for their survival, including collecting bamboo, thatch, fishing, trapping, and other activities [24]. Ginger (*Zingiber officinal*e), maize (*Zea mays*), mustard (*Brassica juncea*), and rice (*Oryza sativa*) are the principal cultivable crops. Cultivating is practiced using both Jhum and settled methods [25]. The Mishings comprise up 79% of the households, while about 21% are Adi Hinduism is practiced by 99% of the households, and Christianity is practiced by 1%. Almost all of the households rely on agriculture and livestock for survival.

The tribe’s indigenous knowledge system displays numerous traditional practices vital for the long-term sustainability and management of forest resources. These include a variety of beliefs that are critical for the safeguarding of sacred groves, where plant material exploitation is prohibited. Festivities and trees are inextricably linked. They have, e.g., a yearly ritual of hunting after worshiping the forest God. During their hunts, these hunters spare both pregnant and young animals. Herbalists in the community do not promote using excessive amounts of therapeutic plants or encouraging their overharvesting, burning, or grazing. Only the essential plant/tree parts are harvested from nearby woodlands. These people have thus established a sustainable connection with the local forest ecosystem. Tree cutting, conversion of forest to agricultural land (Jhum farming), excessive domestic animal grazing, usage of firewood and timber for residential and commercial purposes, fishing, and hunting are all damaging and affecting the corridor’s overall environment [26]. Due to the high pace of population increase, dependency on the forests is growing as well, surpassing the level of exploitation almost a decade earlier [27]. When combined with socio-political alterations within the regions, changes in the effective size, biological flows into and out of the corridor, and increasing exposure to human pressures and invasive species have an impact on the forested landscape [28].

### Methods

During 2022, we collected data from the three villages that are part of the Dering-Dibru Elephant Corridor, located in the Tinali circle in Lower Dibang Valley. In order to carry restoration work successfully, we frequently visited the local communities to develop an understanding and become familiar with their social structure and cultural beliefs. Prior to each interview, verbal prior informed consent was obtained, and the International Society of Ethnobiology Code of Ethics [29] and the International Standards for the practice Society for Ecological Restoration (SER) were followed [30]. The information was gathered via semi-structured questionnaires, complemented by free interviews, group discussions, and direct observations [31–33]. Using a snowball technique, a total of 35 respondents of different ages were selected (Table 1). Participants were asked about the most frequent woody tree species in the nearby forest and which ones are regularly used. The information covers the local name, traditional uses such as food, fodder, timber, fuelwood, medicinal, and species that provide revenue. Participants were also interviewed about the woody tree species used by wild animals for habitat and a food source. The data also cover tree species that grew naturally or were planted in degraded land after Jhum farming. During data collection, a minimum of one knowledgeable informant assisted with plant identification and verification. In order to identify and obtain the native names of the plants, participants were shown photographs as well as live plants. To remove errors and omissions, results were redisplayed to the respondents. The information was acquired in the local language before being translated into English. The selected respondents were mostly illiterate with diverse occupations. They cultivate in both terrace and wet forms, using both Jhum and settled methods. The livelihood is mostly agriculture and allied services. Following the free listing interviews, a tree species checklist was produced. To collect ecological data on woody tree species, a random point sampling technique was used. The points were established using randomization to guarantee that tree species in various habitats had an equal chance of being sampled. For each tree species, the habitat types were documented. The documented tree species were classified into different habitat categories, including natural woodland, riverine, degraded, cultivated field, and roadside.

**Table 1:** Demography of respondents interviewed during the survey

Demographic features	Number	Percentage
Total informants	35	
Gender		
Male	21	60
Female	14	40
Number of villages surveyed	03 (Mer, Paglam, and Namsing)	
Religion	Hinduism and Christianity	
Tribes	Mishing and Aditribe	
Education		
Illiterate	21	60
Primary education	10	28.57
Secondary education	4	11.42
Age		
25–35	2	5.71
36–50	10	28.57
51–75	23	65.71
Professional groups		
Farmers	19	54.28
Herders	7	20.00
Daily wage laborers	5	14.28
Shopkeepers	4	11.42
Socio-economic status	Agriculture and allied services	

**Fig. 2:**
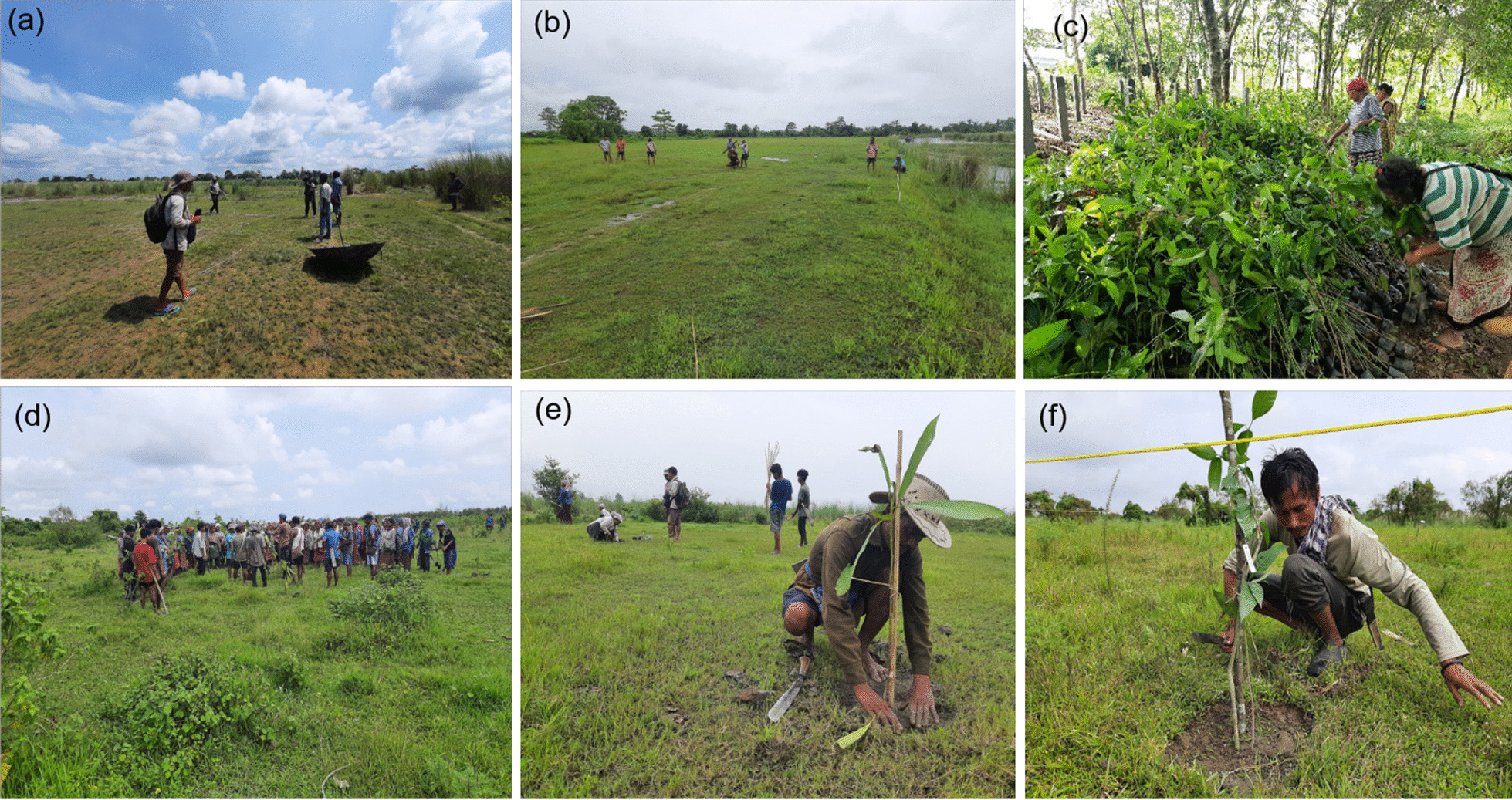
The representative photographs of various activities carried out during the restoration. **a** Land selection, **b** land preparation, **c** community members involvement in taking care of saplings, **d** direct field-based training to local community members at the restoration site, **e** local community members involved in planting saplings, and **f** set permanent plots and tagging saplings for future monitoring

Throughout the restoration process, we consulted the local communities at every stage. Beginning with the selection of tree species, land identification, preparation, planting of saplings, to monitoring and evaluating restoration efficacy, and indigenous tribe communities completed and carried out the restoration work (Fig. 2). To ensure the success of the restoration effort and to advance the villagers’ knowledge of planting saplings, we conducted both theoretical and direct field-based training sessions for them. During the restoration work, 690 individuals (385 males and 305 females) from the local community were trained on the scientific restoration process, such as how to plant the sapling, what is the ideal distance between the saplings, what the size of the pit would be, and how to use bamboo sticks as support for the saplings. In addition, the participants were compensated on a daily basis to improve their living circumstances. We also promote gender equality by involving women in restoration efforts. The restoration area was hand-weeded twice to get rid of undesired plants (especially alien *Mikania micrantha* Kunth, *Mimosa pudica* L., and *Ageratum conizoides* L.). To mitigate the threat posed by excessive domestic animal grazing, we formed a committee to protect these restoration areas through the Village Conservation Forum.

### Use value

UV is used to calculate a species’ relative value in comparison with other species and is calculated as *UV* = *U*/*N* [34] where *U* = number of use reports for a particular species and *N* = total number of informants. A high UV score indicates that the tree species has a large number of usage reports, where as a low UV score indicates that the informants found fewer use reports for that tree species. The value can be anywhere between 0 and 1. Tree species with the most use reports have the highest use value, while those with the few have the lowest value. We employed the fidelity level (FL) to determine which species were the most popular among residents in a given area.

### Data analysis

Principal component analysis (PCA), heat maps, and chord diagrams were used to analyze the data [35]. Our multi-dimensional data were analyzed using PCA to identify hypothetical variables (components) that could explain as much variance as feasible. To do that, we estimated the singular valued composition of the (centered and possibly scaled) data matrix using a matrix of presence/absence in each of the three towns under study. PCA was carried out using R Studio 4.0.1 software. The presence/absence of data was employed in the heat map to indicate the species distribution in the specific clusters due to the same supplying services. The Sorensen’s (Bray–Curtis) distance was employed to find significant differences among various supplying services and resemblances [36]. The program circlize package [37] was used to construct chord diagrams in R software 4.0.1. [38]. Based on the thickness of each bar, we may determine which tree species are associated with which traditional ecological usages, and how many of each species exist in each category [39]. Overall trends in the fidelity level (FL) and used value (UV) were expressed illustratively through linear regression models through GraphPad Prism version 9 (GraphPad Software, CA, USA) [40].

## Results and discussion

### Woody species composition and distribution

**Fig. 3:**
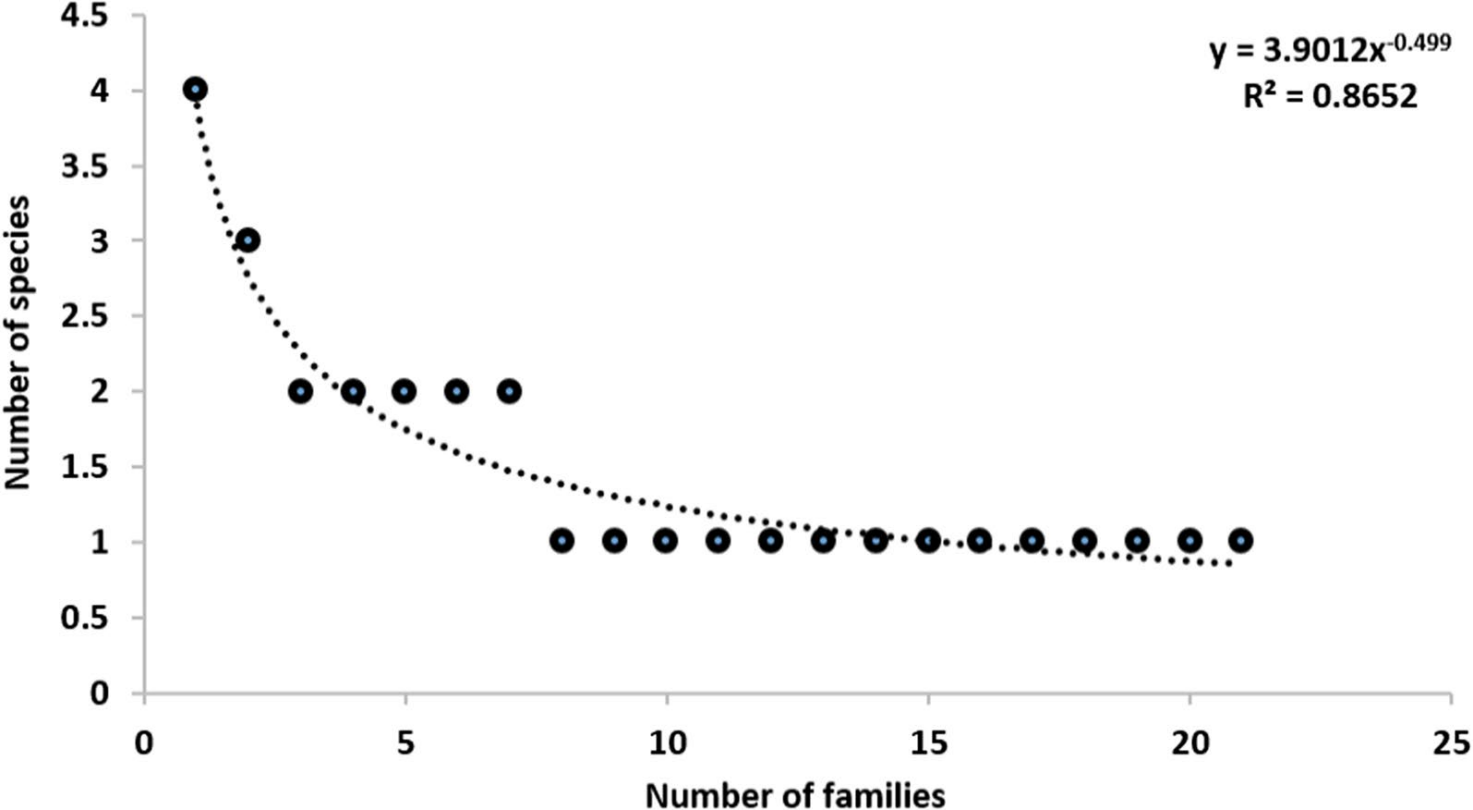
Relationship between families and species pattern in the Dering-Dibru Saikhowa Elephant Corridor in Arunachal Pradesh, India

We planted 95,582 saplings of 31 tree species representing 27 genera and 20 families on 150 hectares of degraded habitat in the Dering-Dibru Saikhowa Elephant Corridor (Table 2). Each woody species’ relevant traditional ecological knowledge was recorded, including family, local name, habitat, citation, and use value (Table 2). The families with the most species having traditional ecological uses were Combretaceae (four species) followed by Fabaceae (three species) and Anacardiaceae (two species) (Table 2). A clear relationship (*y* = 3.9012 × − 0.499; *R*^2^ = 0.8652) between family and species pattern was observed (Fig. 3). The species diversity was similar to other parts of the wider region. Elliott et al. [41] evaluated the potential of 34 tree species for forest restoration in China. Similarly, [42] employed 37 forest tree species to accelerate forest restoration in Doi Suthep-Pui National Park, Thailand. Krishnamurthy et al. [43] similarly reported Combretaceae as the dominant family in Bhadra Wildlife Sanctuary, Karnataka, India. Sen et al. [44] also found Combretaceae as the dominant family in the forests of Dadra and Nagar Haveli, India.

**Table 2:** List of tree species with family, species, local name, habitat and ethno usage, citation, and use value

Family	Species (Abbreviation)	Local name	Habitat	*Ethno usage								Citation	Use value
				F	FO	TI	FW	M	HF	LH	PI		
Apocynaceae	*Alstonia scholaris* (L.)R.Br (Als.sch)	Satiana/Shimol/Shoti	Degraded habitat Natural forests	×	√	×	×	×	×	√	√	20	0.57
Anacardiaceae	*Mangifera indica* L. (Man.ind)	Aam	Riverine Degraded habitat	√	√	×	×	×	√	√	√	16	0.46
	*Spondias mombin* L. (Spo.mom)	Amari/Ugyum	Degraded habitat	√	×	×	×	×	×	×	×	7	0.21
Burseraceae	*Canarium strictum* Roxb. (Can.str)	Dhuna	Natural forests	√	√	×	×	×	×	×	√	11	0.31
Calophyllaceae	*Mesua ferrea* L. (Mes.fer)	Nahar	Degraded habitat Riverine	×	×	√	×	×	×	×	×	13	0.37
Combretaceae	*Terminalia arjuna* Roxb. Ex DC (Ter.arj)	Arjun	Riverine	×	×	×	×	√	×	×	×	14	0.41
	*Terminalia bellirica* (Gaertn.) Roxb. (Ter.bel)	Bhomora/Rotke	Degraded habitat Natural forests	×	×	√	√	√	×	×	×	19	0.54
	*Terminalia chebula* Retz. (Ter.che)	Hilikha	Natural forests Degraded habitat	√	×	×	×	√	×	√	√	22	0.63
	*Terminalia myriocarpa* VanHeurck and Müll.-Arg (Ter.myr)	Halak	Degraded habitat Natural forest	×	√	√	×	×	×	√	×	16	0.46
Dilleniaceae	*Dillenia indica* L. (Dil.ind)	Outenga	Degraded habitat Natural forests Cultivated fields	√	√	×	×	×	√	×	√	20	0.57
Elaeocarpaceae	*Elaeocarpus serratus* L. (Ela.ser)	Jalphai	Degraded habitat Natural forests	√	√	×	×	×	×	√	√	20	0.57
	*Mallotus nudiflorus* (L.) Kulju and Welzen (Mal.nud)	Bhelkor	Roadside Natural forests Cultivated fields	×	×	×	√	×	×	×	×	10	0.29
Fabaceae	*Albizia lebbeck* (L.) Benth (Alb.leb)	Sirish	Riverine Degraded habitat	×	√	√	×	×	√	×	√	17	0.49
	*Dalbergia sissoo* Roxb. Ex DC (Dal.sis)	Sissoo	Degraded habitat Roadside Natural forests	×	×	√	√	×	×	√	×	18	0.51
	*Samanea saman* (Jacq.) Merr (Sam.sam)	Raintree/tantari	Degraded habitat Natural forests	×	×	√	√	×	√	√	√	13	0.37
Lamiaceae	*Gmelina arborea* Roxb. Ex Sm (Gme.arb)	Gamari	Riverine Natural forests Cultivated fields	×	×	√	√	×	√	×	√	17	0.49
Lythraceae	*Lagerstroemia speciosa* (L.) Pers (Lag.spe)	Azar	Natural forests	×	×	√	√	×	×	×	√	19	0.54
Malvaceae	*Bombax ceiba* L. (Bom.cei)	Simalu/Sing-ii	Degraded habitat Riverine	×	×	√	√	×	√	√	√	23	0.66
Magnoliaceae	*Magnolia baillonii* Pierre (Mag.bai)	Titachapa	Degraded habitat Natural forests	×	×	√	√	×	×	×	×	16	0.46
Meliaceae	*Chukrasia tabularis* A.Juss (Chu.tab)	Bogipoma/Shilling/Ketem	Degraded habitat Natural forests	×	×	√	√	×	×	√	×	13	0.37
	*Azadirachta indica* A.Juss (Aza.ind)	Neem	Degraded habitat Cultivated fields	√	×	√	√	√	√	×	√	21	0.61
Moraceae	*Artocarpus chama* Buch.-Ham (Art.cha)	Chamkothal	Degraded habitat Natural forests Cultivated fields	√	×	×	×	×	×	√	×	19	0.54
	*Artocarpus heterophyllus* Lam. (Art.het)	Kothal	Degraded habitat Cultivated fields	√	√	×	×	×	√	√	√	26	0.74
Myrtaceae	*Psidium guajava* L. (Psi.gua)	Guava	Degraded habitat Cultivated fields Roadside	√	√	×	×	√	√	√	√	14	0.41
	*Syzygium cumini* (L.) Skeels (Syz.cum)	Jam	Degraded habitat Riverine	√	√	×	×	√	√	√	√	16	0.46
Oleaceae	*Chionanthus ramiflorus* Roxb. (Chi.ram)	Ketiri momiri	Natural forests	×	×	×	×	×	√	×	×	9	0.26
Oxalidaceae	*Averrhoa carambola* L. (Ave.car)	Kordoi	Degraded habitat Natural forests Riverine	√	√	×	×	×	√	√	√	10	0.29
Phyllanthaceae	*Bischofia javanica* Blume (Bis.jav)	Urium/Takkir/Sheetal	Natural forests Riverine Degraded habitat	×	×	×	√	×	√	×	×	25	0.71
	*Phyllanthus emblica* L. (Phy.emb)	Aamlakhi	Degraded habitat Cultivated fields Natural forests	√	√	×	×	√	√	√	√	22	0.63
Rubiaceae	*Neolamarckia cadamba* (Roxb.) Bosser (Neo.cad)	Kadam	Degraded habitat Natural forests Riverine	×	×	√	√	×	×	×	×	19	0.54
Sapotaceae	*Manilkara kauki* (L.) Dubard (Man.kau)	Bokul	Riverine Natural forests	√	×	×	×	×	×	×	×	19	0.54

*F (food), FO (fodder), TI (timber), FW (fuelwood), M (medicine), HF (habitat and forage), LH (livelihood), and PI (pioneer)

### Traditional knowledge on enlisted species

**Fig. 4:**
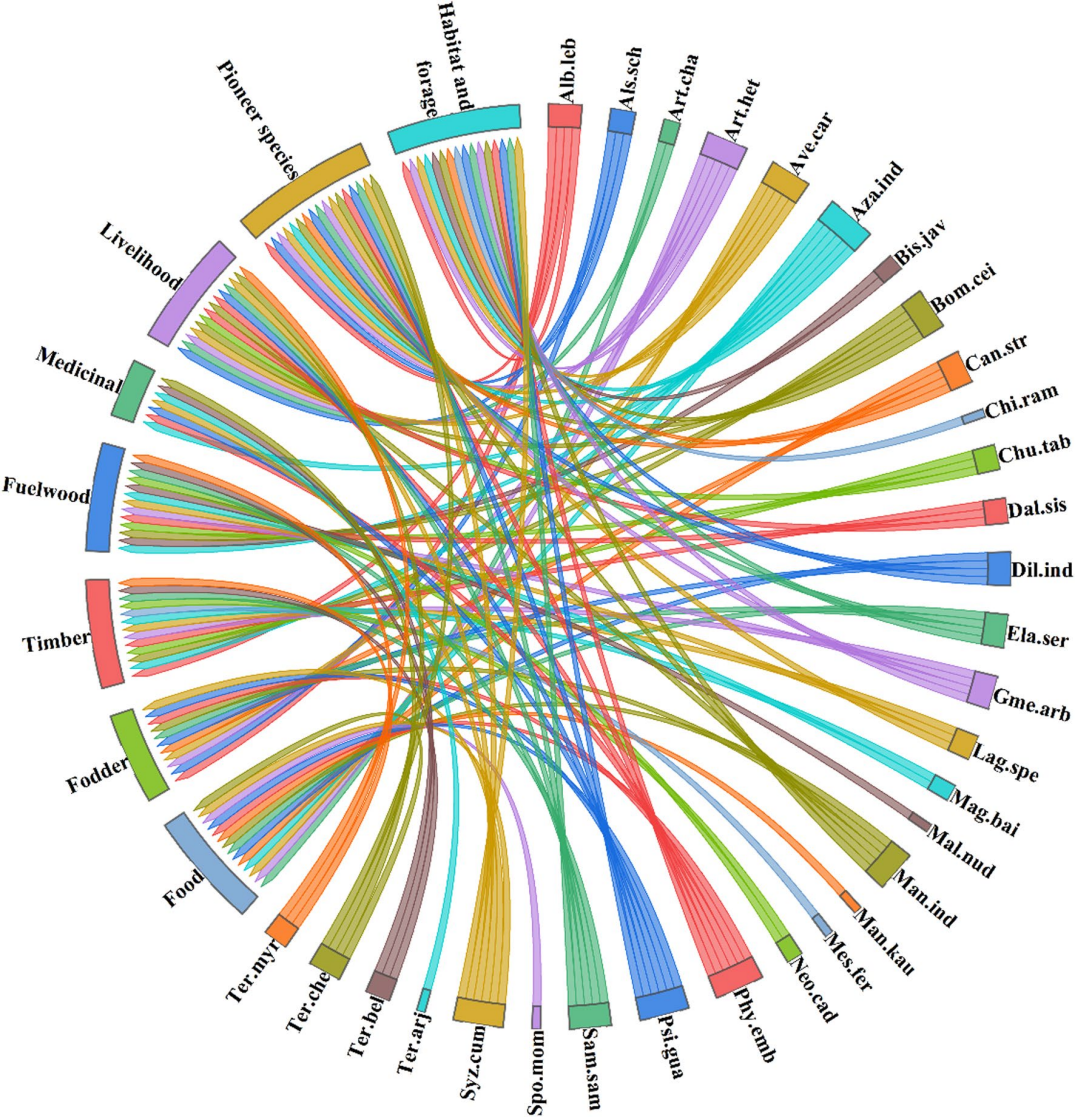
Woody species distribution according to usage by native communities’ in Dering-Dibru Saikhowa Elephant Corridor in Arunachal Pradesh, India. The direction of the lines shows which woody species are associated with which types of usage, and the thickness of each bar shows the number of species in each usage category. The complete name of species is shown in Table 2

We classified the documented species based on the ecological knowledge of the local inhabitants, i.e., as pioneer species, food, fodder, timber, fuelwood, medicinal, livelihood, habitat, and forage. Upon interpreting the documented results, pioneer species accounted for 16% of all woody species, followed by habitat and forage for wildlife (15%), food, timber, and livelihood species (13% each), fuelwood (12%), fodder (11%), and medicine (7%) (Fig. 4 and Table 2). De Arruda et al. [45] reported the dominance of ethno-woody species as pioneer species in forests of Northeast Brazil. For indigenous people who live in or close to forests, non-timber forest products have grown to be a significant source of revenue and sustenance. Such traditional conservation of woody and related plants serves as a “safety net” and “resource ground” for the community as well as helping to preserve biodiversity and natural resources [46].

**Fig. 5:**
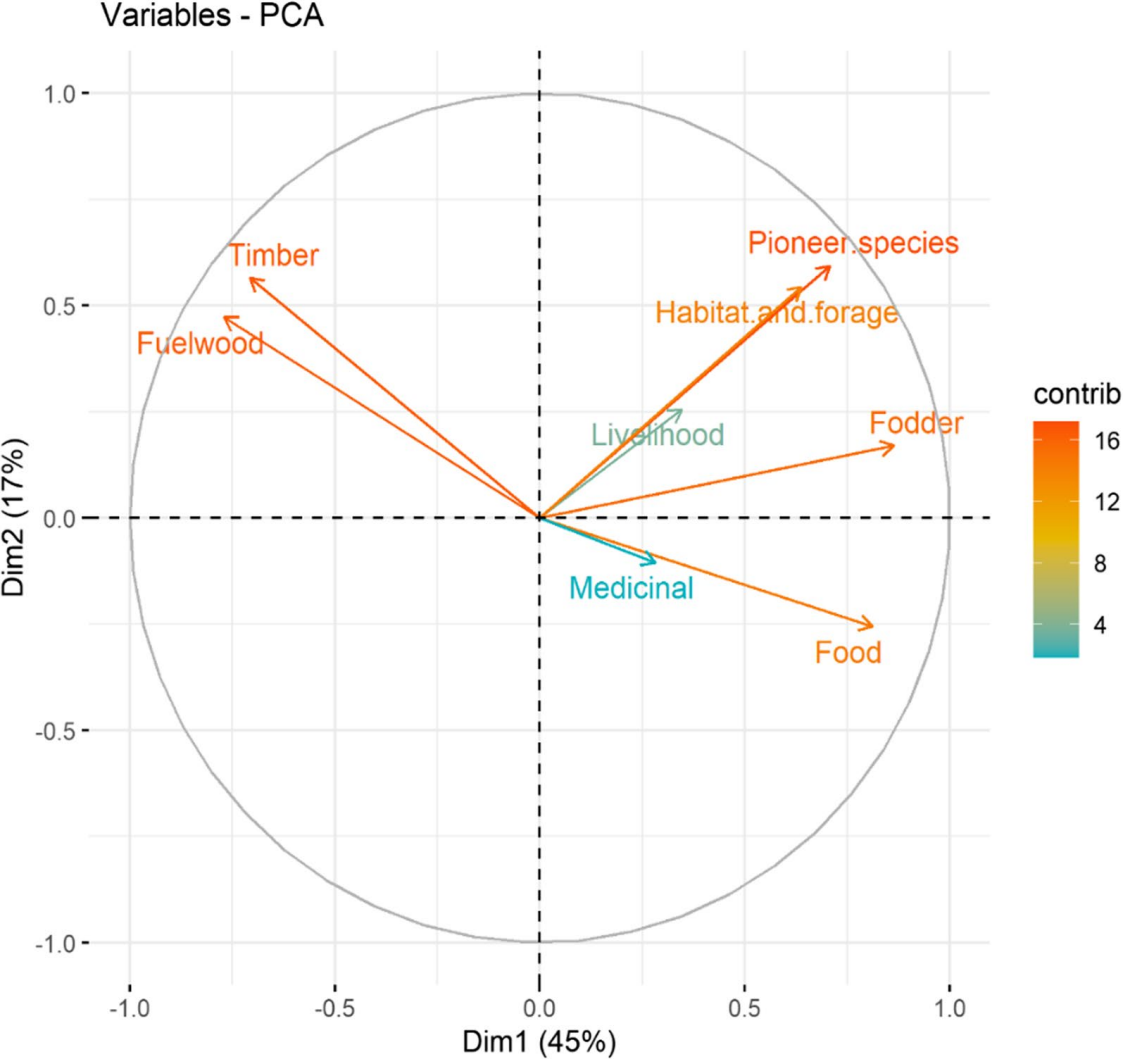
Principal component analysis (PCA) illustrating the relationship of woody species and the usage by native communities’ in Dering-Dibru Saikhowa Elephant Corridor in Arunachal Pradesh, India

This result was further verified by PCA analysis (Fig. 4), which yielded separate groups depending on preferences for woody species utilization. The two axes accounted for 62% of the variation in the biplot. PC1 accounted for 45% of the variation in species utilization, with PC2 accounted for the remaining 17%. The individual uses, i.e., food, fodder, timber, fuelwood, pioneer species, habitat, and forage closest to 1 (positive) or -1 (negative) have the greatest correlations, whereas medicinal and livelihood show less correlation. Timber and fuelwood were clearly segregated on the one side of the PCA from the rest of the uses on the other (Fig. 5). In a similar manner, [47–49] employed multivariate analysis in TEK and ecological restoration.

### Woody vegetation usage patterns

**Fig. 6:**
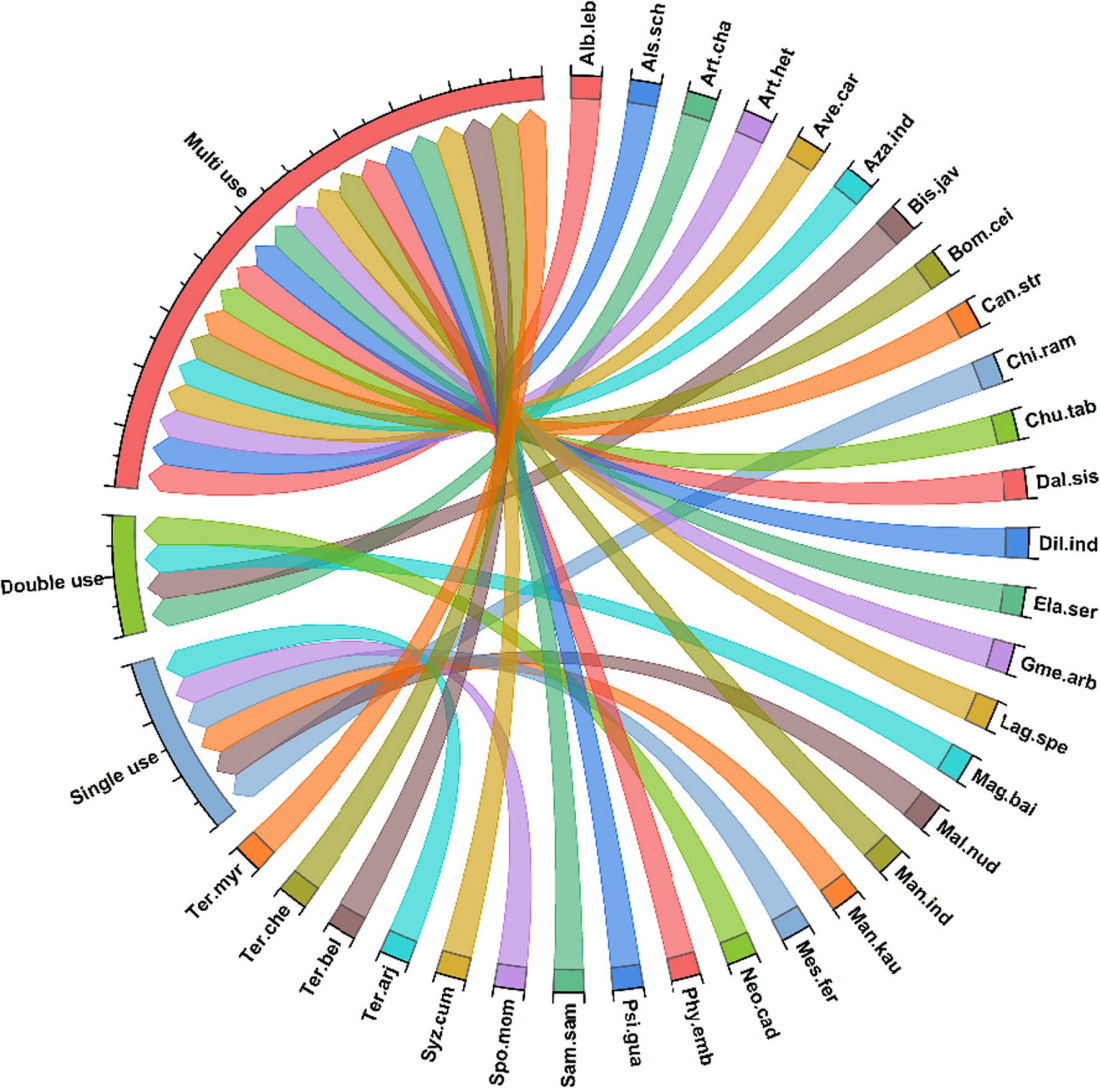
Woody species and their usage (single, double, and multi) in Dering-Dibru Saikhowa Elephant Corridor in Arunachal Pradesh, India. The direction of the lines shows which woody species are associated with which types of usage patterns, and the thickness of each bar shows the number of species in each category. The complete name of species is shown in Table 2

During the present study, the ecological usage pattern of woody species across the local inhabitants was categorized into single, double, or multi-uses (Fig. 6). We found ten species with more than five applications, with *Azadirachta indica, Phyllanthus emblica,* and *Syzygium cumini* (six each) having the greatest use value, and *Mesua ferrea* having the lowest number of uses (single use).

#### Single use

These were the woody species used for only single purpose, including *Chionanthus ramiflorus*, *Mallotus nudiflorus, Manilkara kauki, and Mesua ferrea.* The single usage species constituted 19% of the total used species.

#### Double use

Woody species used for two purposes were *Artocarpus chama, Bischofia javanica, Magnolia baillonii, and Neolamarckia cadamba.* Double uses species constituted 13%.

#### Multi-use

**Fig. 7:**
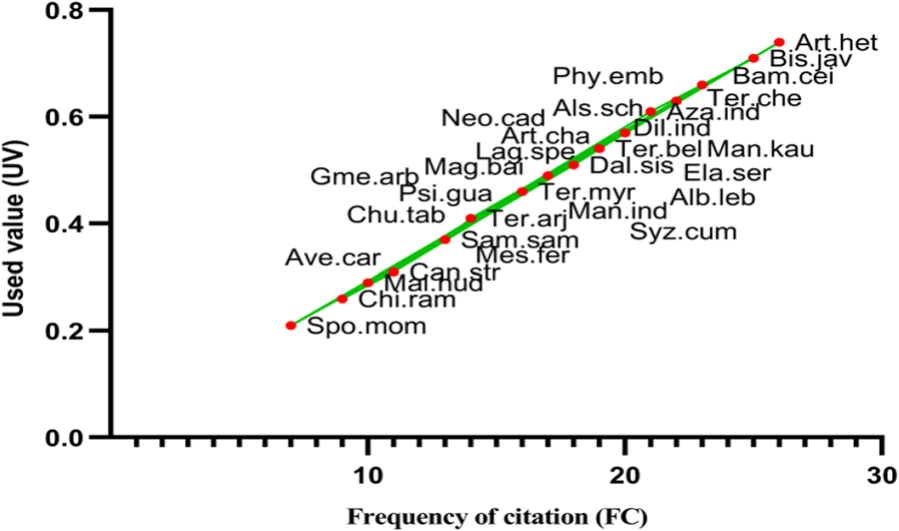
Relationship between used value (UV) and frequency of citation (FC). The plant names as they appear in Table 2

Woody species such as *Albizia lebbeck, Artocarpus heterophyllus, Azadirachta indica, Bombax ceiba, Dalbergia sissoo, Dillenia indica, Lagerstroemia speciosa, Phyllanthus emblica, Syzygium cumini, and Terminalia chebula* were used for multiple purposes. Multi-use species constituted 68% of all species. We employed the use value (UV) index to elucidate a relation between each species and the uses allocated to them. The UVs of different species are given in Fig. 7. Among the reported species, the highest use value was calculated for *Artocarpus heterophyllus* (*UV* = 0.74) and the lowest for *Spondias mombin* (*UV* = 0.2). The high use value (*UV*) of the *Artocarpus heterophyllus* was due to its multiple usage as well as the familiarity local people in its usage since ancestor times (Fig. 7). According to [50], the use of plant species highly depends upon social factors and differs across locations. Such patterns have been observed around the globe, e.g., Mariscal et al. [51] found the number of uses of a species ranging from one to a maximum of five in Cloud Forest Ecosystem Restoration in Ecuador, Guariguata et al. [52] reported different usage patterns of species for forest restoration from Costa Rica. Similarly, [53] reported a variety of species applications from tropical montane forests. According to [54], there is abundant TEK existent in the Arunachal Himalayan hill communities. This knowledge, which has been adapted to the local ecosystem and is valuable for sustainable resource use and conservation, is based on millennia of spontaneous observations of the local environment. Hazarika and Merentoshi [55] reported the maximum use value of *Artocarpus heterophyllus* from Nagaland, India. Similarly, [56] reported the same while investigating the traditional agricultural practices in Meghalaya, India.

### Ecological knowledge on enlisted species

In the present study, we found that the local people were quite knowledgeable and provided detailed information on species required as pioneer store a variety of habitats (Table 2). It is important to mention that local people use forestland for agriculture, for which they burn the vegetation on a patch of land, which, in turn, increases the fertility of land. The obtained land is used for 3–4 years, after which a second patch is selected, and TEK is applied to restore the left behind patch rapidly. We incorporated this traditional ecological knowledge into ecological restoration programs by planting mixed pioneer tree species. An overall of 16% of species including *Bombax ceiba, Albizia lebbeck, Dillenia indica, Syzygium cumini, Phyllanthus emblica, Lagerstroemia speciosa, Alstonia scholaris, and Averrhoa carambola* were reintroduced to restore the degraded habitats. Throughout the world, local people possess an extensive traditional ecological knowledge of the indigenous species with relation to habitat [57]. The traditional knowledge and well-adapted traditional agricultural techniques of the indigenous communities allowed them to maintain an ecological balance [58].

**Fig. 8:**
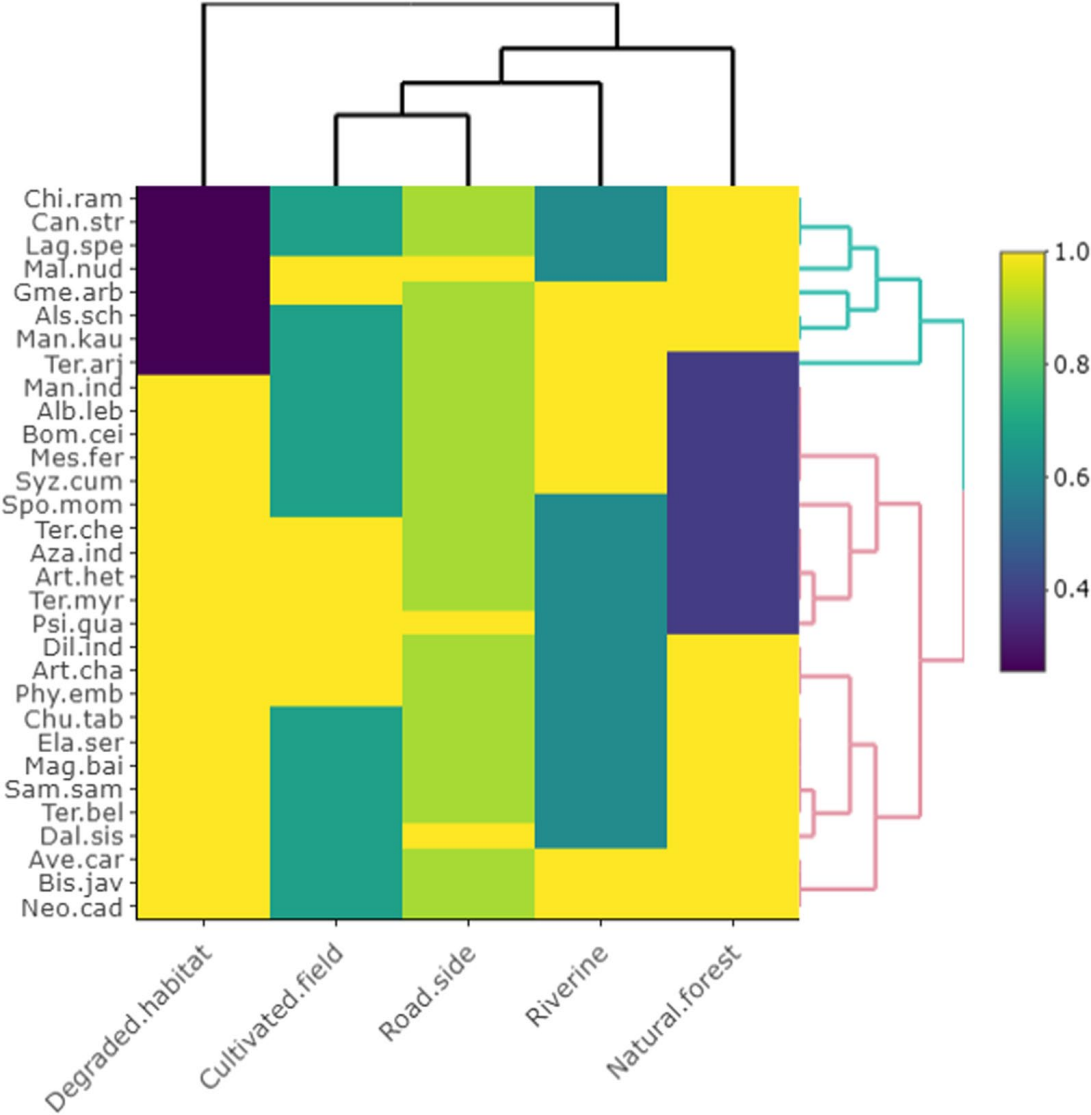
Heat map illustrating the relationship of woody species and the habitat types in Dering-Dibru Saikhowa Elephant Corridor in Arunachal Pradesh, India

We classified the habitat of enlisted species into different categories including, natural forest, riverine, roadside, cultivated field, and degraded habitat. Based on the similarity of woody species in different habitats, two primary clusters (clusters 1 and 2) were obtained (Fig. 8). Cluster 1 included eight species that grew mostly in degraded habitat. Cluster 2 included two branches displaying the classified habitats such as natural forest, riverine, roadside, and cultivated field. However, the species that are closely grouped in close clusters are more similar in habitat preference. Most of the species grew in more than one habitat (see Table 2 for habitat preferences). Our results are in line with [59] from Kerala, [60] from southeastern Mexico, [61] from Madhya Pradesh, and [62] from Himalaya.

**Fig. 9:**
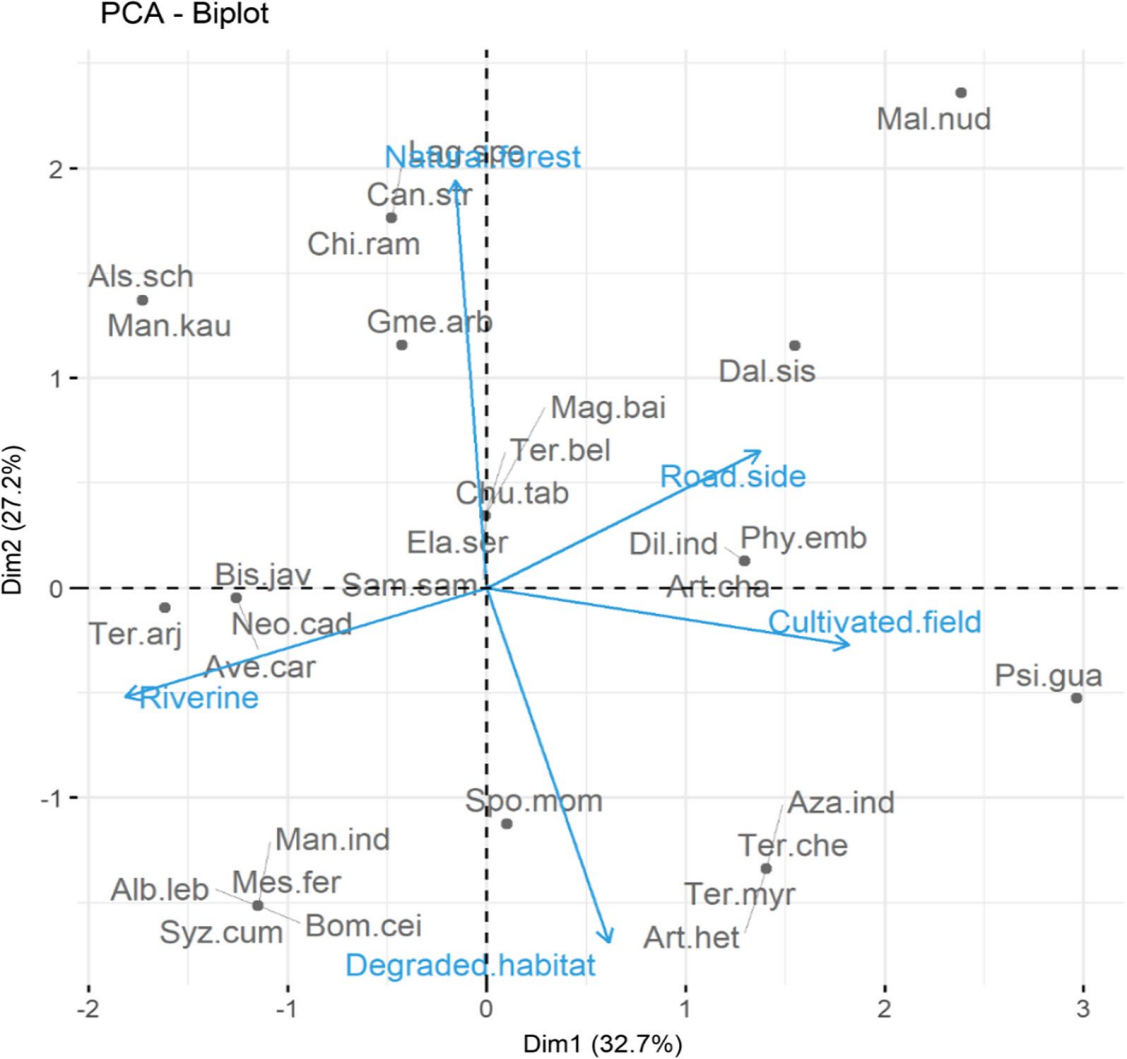
Principal component analysis (PCA) illustrating the relationship of woody species and the habitat types in Dering-Dibru Saikhowa Elephant Corridor in Arunachal Pradesh, India

Our results were further supported by PCA, which showed variation depending on habitat preferences of woody species. The two axes account for 59.9% of the variation in the biplot. PC1 accounts for 32.7% of the variation in species habitat preference, with PC2 accounting for the remaining 27.2% (Fig. 9). The habitat types, i.e., natural forest, riverine, degraded, and cultivated field closest to 1 (positive) or -1 (negative) had the greatest correlations. Our study found that tribal people based on their surrounding environment recognized woody species. One example is species such as *Lagerstroemia speciosa, Chionanthus ramiflorus, Canarium strictum, Gmelina arborea, and Manilkara kauki* that were regarded as the indicator species for natural forest. Subashree et al. [63] found *Chionanthus ramiflorus and Canarium strictum* as the key pioneer species in a study on the structure and regeneration potential in Western Ghats. Species such as *Albizia lebbeck, Bombax ceiba, Syzygium cumini, Spondias mombin, Mesua ferrea, and Artocarpus heterophyllum* were associated the degraded habitats. Similarly, [64] reported *Albizia lebbeck* and *Bombax ceiba*as characteristic for degraded habitats. Riverine habitat included species such as *Artocarpus chama, Bischofia javanica, Samanea saman, and Neolamarckia cadamba*. Similarly, [65] reported *Artocarpus chama and Bischofia javanica* as the major riverine species from Assam, India. Roadside habitat included species such as *Dillenia indica, Phyllanthus emblica, and Dalbergia sissoo*, and the cultivated field habitat included *Psidium guajava* and *Azadirachta indica*, and [66] also reported *Dillenia indica* in roadside and cultivated field habitats from Northeast India.

Among the species were introduced to the corridor, 69% were tied to indigenous societies through ethno uses as food, timber, fuelwood, medicine, and fodder, thus integrating resource use patterns and societal and cultural values in restoration design is a significant goal. *Artocarpus heterophyllus*, *Artocarpus chama*, *Albizia lebbeck*, *Bombax ceiba*, *Canarium strictum*, *Chukrasia tabularis*, *Dalbergia sissoo*, *Elaeocarpus serratus*, *Gmelina arborea*, *Magnolia baillonii*, *Mangifera indica*, *Neolamarckia cadamba*, *Samanea saman*, *Terminalia chebula*, and *Terminalia myriocarpa* were used by native communities for food, timber, and fuelwood. Intensive afforestation/reforestation based on planting native tree species offer a wide range of ecosystem services for the local community. Local peoples’ knowledge of plant species is an important source of information on species distribution, rarity, ethno usage, and long-term vegetation change [67]. In northeastern India, tribal people share vest a variety of forest products from privately owned and community-maintained woods for a variety of uses [68]. In this setting, *Lagerstroemia speciosa* and *Mesua ferrea* were considered the best fuelwood plants. Similarly, *Azadirachta indica*, *Psidium guajava*, *Terminalia arjuna*, *Terminalia chebula*, and *Terminalia arjuna* were also of medicinal importance and were reintroduced given that the local native communities still rely on these natural resources for their health care. Kiran et al. [69] reported *Terminalia arjuna* as medicinally important species utilized for reclaiming degraded land in India. Similarly, [70] reported species like *Azadirachta indica* for conservation and restoration of degraded terrestrial ecosystem in Central India. These diverse plantings represent a system-level move away from monoculture plantations in order to increase biodiversity and aid in the recovery of ecosystem functioning. Planting multiple tree species frequently includes legumes, i.e., *Albizia lebbeck* that fix nitrogen; this nitrogen supply, together with litter from vegetation, reduces or eliminates the need for additional fertilizer. Forage plants such as *Dillenia indica and Elaeocarpus serratus* can also be grown to provide fuelwood and fruit, enabling sustainable land use. Furthermore, increased soil fertility may reduce farmers’ need to expand their farming activities (Jhum cultivation) into the forest. We must reestablish a healthy relationship with the ecosystems that support us. Restoration is critical to reducing climate change, maintaining food security for a growing population, and reversing biodiversity loss. We also require solutions that are natural, such as restoration.

### Enhancing biodiversity

A total of 15% of the total woody species reported were restored for animal habitat and forage, aiming to achieve the primary goal of restoring biodiversity and functionality of degraded habitats. *Chionanthus ramiflorus, Artocarpus heterophyllus*, and *Dillenia indica* are among the plants valuable to wildlife, providing both food and habitat (perching and nesting grounds) for a variety of birds and animals. Maintaining biodiversity requires a multifaceted concept that includes species richness, and diversity is a priority in the ecological restoration design [71, 72]. A preferred plant species is defined by the extent to which the species is consumed in relation to its availability in the environment [73]. Sathya et al. [74] reported the *Chionanthus ramiflorus and Artocarpus heterophyllus* as the species being exploited by the elephants in Sathyamangalam Tiger Reserve, southern India; elephants prefer the woody species, such as *Bischofia javanica* and *Neolamarckia cadamba*. Mathew [75] also reported the preference of woody species like *Bischofia javanica* by the elephants and other wild animals. Birds and other animals eat and live in woody plants such as *Phyllanthus emblica, Mangifera indica, Psidium guajava, Syzygium cumini, Artocarpus chama, Averrhoa carambola, Elaeocarpus serratus,* and *Manilkara kauki.* Thakur et al. [76] found the use of many foraging species (*Artocarpus heterophyllus*) when investigating the feeding behavior of elephants in Chhattisgarh, India. Land use efficiency could make more space available for wildlife than traditional single-species plantations. Multiple trees are introduced to boost biodiversity at the landscape level by giving wildlife habitat and structural connectivity. If this method, which combines parts of sharing and protecting land, was supported by legislation, it would probably be able to prevent deforestation and the degradation of elephant corridors. It can also be effective in limiting Jhum expansion. We need to halt the loss of natural habitat, preserve what is left, and restore damaged ecosystems if we want to reverse the trend of biodiversity loss [77].

### Community engagement and livelihood

Poverty can worsen damage to ecosystems and is a contributing factor to land degradation [78]. The health of young people and elders, women, the poor, indigenous peoples, those with chronic health conditions, and those who are the targets of racism are all at risk due to degradation, which also adversely affects indigenous and local communities whose livelihoods are directly dependent on natural resources [79, 80]. People in the developing world are highly dependent on woody species [34], from which they generate means for their livelihoods. However, wild trees are often threatened and decline in abundance and diversity. The main reason behind is agricultural expansion and overexploitation [81]. Large size, poor dispersal capacities, and low reproductive rates are the characteristics that render species susceptible to local extinction. At the same time, over exploitation is influenced by cultural and economic factors. They were introduced 13% of the enlisted species as a source of income for the native people, fitting one of the restoration design’s primary objectives. *Artocarpus heterophyllus, Averrhoa carambola, Mangifera indica, Phyllanthus emblica, Psidium guajava,* and *Syzygium cumini* were planted to help the community relying on the natural forest for their livelihood. Rahman et al. [82] reported on similar initiatives from Southeast Bangladesh, where they distributed saplings of a variety of medicinal and fruit tree species and supported the cultivation of medicinal plants in homestead forests and inside a sanctuary, distributed seeds of a range of seasonal vegetables for immediate cash returns, and established a committee through the Village Conservation Forum to protect these plantations. The success of any integrated science-based plan requires good conservation management in the field especially at the local level, and TEK is a step toward a management strategy for ecosystem restoration. An adaptive management plan makes it easier to combine various information sources, educate the public, and launch conservation policies in an iterative process that would develop as well earn how to protect these vulnerable ecosystems. Creating an action plan for forest protection should begin by convening local communities, scientists, resource managers, and government representatives in workshops [83]. Such a strategy would be in accordance with the UN Decade of Ecosystem Restoration (2021–2030), which requires governments across the countries to integrate local communities and their indigenous knowledge with management goals [84].

### Revitalization of local plant knowledge

TEK systems representing cultural diversity are intricately linked to biological diversity, and the loss of either can have a significant effect on the other. If plant knowledge is lost, there is a chance that this will have a domino effect that causes the depletion of natural resources, a loss of biodiversity, and the extinction of plant species. Contrarily, a drop in diversity may also cause a loss or transformation of knowledge in the variety and number of used species. It has also been demonstrated that the continual loss of TEK due to cultural and linguistic extinction undermines conservation efforts. Because of social and ecological changes, the subsequent loss of land tenure, changes in educational practices, traditional livelihoods, and beliefs, as well as the loss of rights, all pose threats to the integrity of TEK on a larger scale [85, 86]. The frequent inability of indigenous people to access and defend their own ancestral lands endangers the survival of TEK given that cultures decline in the absence of suitable environments [87, 88]. Prioritizing indigenous rights may increase the possibility of achieving global conservation goals with positive effects on all life on Earth. Greater co-benefits are also largely predicated when respecting traditional knowledge systems and directly promoting indigenous leadership in restoration initiatives, in addition to rights [89]. Therefore, we suggest a thorough dialog with indigenous peoples to be undertaken in order to considering TEK collaborations in restoration. We further stress that any knowledge exchange must be conducted primarily via careful listening and appropriate engagement in a way that supports indigenous leadership and communication customs, and guards against the degradation of ecological and cultural integrity. Ecological restoration strategies that consider the intertwined features of biodiversity and ecosystem services can only be successful if the people who will benefit from them are considered throughout policy development and implementation.

### Monitoring of restoration success

Traditional ecological knowledge can provide a strong foundation for ecological restoration success because it co-evolved with the natural ecosystem. Techniques for restoration should consider such cultural interactions. We engaged the local community in conservation efforts by documenting traditions and livelihoods that depend on the environment, some of which had only been retained by elders, and then educating community members accordingly. To measure the important long-term ecological characteristics, permanent plots were installed in the restoration sites, and data are continuously gathered during the peak of the growing season each year. Site photographs and data supplied as field notes are included in the documentation. In order to record the total number of species in the restoration sites, including tree species newly regenerated due to our efforts, an annual survey is being carried out in the restoration sites. The annual report also calculates the importance value index for each species, the survival rate of newly planted tree saplings, and the status of quantifiable biodiversity indicators.

### Recommendations

Ethnobiological studies can improve communication between researchers, management plan developers, and local communities, which is crucial for the creation of effective conservation measures. For example, a better grasp of specific knowledge of wildlife can aid in the development of elaborate community-based conservation activities. Because local communities are aware of the paths and locations of the specific species that a visitor or wildlife photographer is hoping to see, the knowledgeable local inhabitants play an important role in tourism development. The influx of national and international tourists seeking to see and photograph elephants and other wild animals can greatly improve the local economy and raise public awareness. The wildlife department and wildlife NGOs like the Wildlife Trust of India (WTI) have supported local communities in wildlife-rich areas through natural-based livelihoods such as homestays, piggery, fishery, and eco-tourism organizations, which help to improve the livelihood of the fringe communities. In addition, wildlife NGOs (WTI) are facilitating the linking of programs like self-help groups where these villages receive loans without interest to start small businesses to empower the women in order to improve local income, reduce dependency, and gain support for wildlife conservation. We believe that more effective knowledge system bridging will raise the likelihood of success and lead to better collaboration between conservation practice, academic science, and indigenous and traditional knowledge holders. Traditional knowledge bearers and their knowledge, in our opinion, can help to promote the protection of species and habitats, stimulate the sustainable use of biodiversity, and raise awareness of the need of conservation more effectively.

## Conclusion

Research on microbial, fungus, and soil ecology sheds light on relatively unexplored ecological processes of enormous scope and directly responds to a need in restoration practice for practical and economical methods for site amelioration. Continued research into the ecology of soil microbes may reveal new potential for increasing restoration success and may provide missing pieces in our understanding of community development. For example, studies of assembly and diversity and function in soil communities have traditionally been overlooked in ecology, and restoration practice must fill in the knowledge gaps. We strongly urge restoration scientists to address these concerns. However, limitations in scientific understanding offer multiple challenges to effective restoration.

Using TEK as a guide, we prioritized the reintroduction of 31 tree species in the degraded forest habitats of the Dering-Dibru Saikhowa Elephant Corridor landscape restoration reference sites. In addition to providing food and other necessities, they also offer habitat for biodiversity, economic opportunities, and advantages for our spiritual and cultural traditions. TEK may aid ecological restoration by assisting in the selection of tree species for planting. Natural ecosystem research and traditional ecological knowledge can thus provide useful information on ecosystem–plant–animal relationships, as well as identify native tree species that benefit humans and animals. This study also demonstrates that TEK can contribute to all aspects of ecological restoration, from the reconstruction of the reference ecosystem and adaptive management to species selection forest oration and monitoring and the evaluation of restoration outcomes. Local ecological expertise can offer insightful opinions on sustainable forest management techniques that have evolved endogenously over many generations in the natural environment. Therefore, revitalizing local ecological knowledge and practices is crucial for the ecological transition since it may encourage sustainable land use practices, enhance biodiversity, and assist and empower local communities.

## Data Availability

All the data are available in manuscript.

## Abbreviations

TEK: Traditional ecological knowledge
F: Food
FO: Fodder
TI: Timber
FW: Fuelwood
M: Medicine
HF: Habitat and forage
LH: Livelihood
PI: Pioneer
PCA: Principal component analysis
FL: Fidelity level
UV: Use value
NEI: Northeast India
UN: United Nations
NGOs: Non-Governmental Organizations

## References

[1] UN Free prior and informed consent—an indigenous peoples’ right and a good practice for local communities—FAO. https://www.un.org/development/desa/indigenouspeoples/publications/2016/10/freepriorand-informed-consent-an-indigenous-peoples-right-and-a-good-practicefor-localcommunities-fao/. Accessed 18 Jan 2021.

[2] Robinson JM, Gellie N, MacCarthy D, Mills JG, O’Donnell K, Redvers N (2021). Traditional ecological knowledge in restoration ecology: a call to listen deeply, to engage with, and respect Indigenous voices. Restor Ecol.

[3] Tengö M, Brondizio ES, Elmqvist T, Malmer P, Spierenburg M (2014). Connecting diverse knowledge systems for enhanced ecosystem governance: the multiple evidence base approach. Ambio.

[4] Reyes-García V, Fernández-Llamazares Á, McElwee P, Molnár Z, Öllerer K, Wilson SJ, Brondizio ES (2019). The contributions of Indigenous Peoples and local communities to ecological restoration. Restor Ecol.

[5] Fritz S, See L, Carlson T, Haklay MM, Oliver JL, Fraisl D, Mondardini R, Brocklehurst M, Shanley LA, Schade S, Wehn U (2019). Citizen science and the United Nations sustainable development goals. Nat Sustain.

[6] Bethel MB, Braud DH, Lambeth T, Dardar DS, Ferguson-Bohnee P (2022). Mapping risk factors to climate change impacts using traditional ecological knowledge to support adaptation planning with a Native American Tribe in Louisiana. J Environ Manag.

[7] Fischer J, Riechers M, Loos J, Martin-Lopez B, Temperton VM (2021). Making the UN decade on ecosystem restoration a social-ecological endeavour. Tren Ecol Evolut.

[8] Snowball J, Collins A, Nwauche E (2021). Ethics, values and legality in the restoration of cultural artefacts: the case of South Africa. Int J Cult Pol.

[9] Zidny R, Sjöström J, Eilks I (2020). A multi-perspective reflection on how indigenous knowledge and related ideas can improve science education for sustainability. Sci Educat.

[10] Uprety Y, Asselin H, Bergeron Y, Doyon F, Boucher JF (2012). Contribution of traditional knowledge to ecological restoration: practices and applications. Ecoscience.

[11] Gutierrez V, Hallett JG, Ota L, Sterling E, Wilson SJ, Bodin B, Chazdon RL (2022). Forest and landscape restoration monitoring frameworks: how principled are they?. Restor Ecol.

[12] Temperton VM, Buchmann N, Buisson E, Durigan G, Kazmierczak Ł, Perring MP, de Sá DM, Veldman JW, Overbeck GE (2019). Step back from the forest and step up to the Bonn Challenge: how a broad ecological perspective can promote successful landscape restoration. Restor Ecol.

[13] Fox H, Cundill G (2018). Towards increased community-engaged ecological restoration: a review of current practice and future directions. Ecolog Restorat.

[14] Svenning JC, Munk M, Schweiger A (2019). Trophic rewilding: ecological restoration of top-down trophic interactions to promote self-regulating biodiverse ecosystems. Rewilding.

[15] Ogar E, Pecl G, Mustonen T (2020). Science must embrace traditional and indigenous knowledge to solve our biodiversity crisis. One Earth.

[16] Adade Williams P, Sikutshwa L, Shackleton S (2020). Acknowledging indigenous and local knowledge to facilitate collaboration in landscape approaches—lessons from a systematic review. Land.

[17] Molnár Z, Babai D (2021). Inviting ecologists to delve deeper into traditional ecological knowledge. Tre Ecol Evolut.

[18] Ramakrishnan PS (2007). Traditional forest knowledge and sustainable forestry: a north-east India perspective. Forest Ecol Manag.

[19] Pandey DK, De HK, Dubey SK, Kumar B, Dobhal S, Adhiguru P (2020). Indigenous people’s attachment to shifting cultivation in the Eastern Himalayas, India: a cross-sectional evidence. Forest Pol Econ.

[20] Das P, Behera MD, Barik SK, Mudi S, Jagadish B, Sarkar S, Joshi SR, Adhikari D, Behera SK, Sarma K, Srivastava PK (2022). Shifting cultivation induced burn area dynamics using ensemble approach in Northeast India. Tre For Peop.

[21] Janaki M, Pandit R, Sharma RK (2021). The role of traditional belief systems in conserving biological diversity in the Eastern Himalaya Eco-region of India. Human Dimen Wild.

[22] Baidya S, Bijay T, Ashalata D (2020). Ethnomedicinal plants of the sacred groves and their uses by Karbi tribe in Karbi Anglong district of Assam, Northeast India. Ind J Tradit Knowl.

[23] Jeyaprakash K, Rathinavel S (2016). Floristic investigation on D’Ering memorial wildlife sanctuary, Arunachal Pradesh, Eastern Himalaya. India Int J Res Plant Sci.

[24] Grewal DS. Tribes of Arunachal Pradesh identity, culture and languages. 2011.

[25] Modi P (2020). Commercial ginger cultivation and its socio-economic contribution-empirical evidences from Lohit and Lower Dibang Valley Districts of Arunachal Pradesh.

[26] Nongmaithem R, Lodhi MS, Samal PK, Dhyani PP, Sharma S (2016). Faunal diversity and threats of the Dibru-Saikhowa biosphere reserve: a study from Assam, India. Int J Conserv Sci.

[27] Deka G. Mode of human interaction and adjustment to the forest environment: a case study in Dibrusaikhowa national park, Tinsukia, Assam. In: Proceeding of ICHR sponsored national seminar on ’Relationship between the environment awareness and its conservation during Vedic period and of present time’organizd by Koba PG College, Dariyapur, Azamgarh. 2012.

[28] Shah RK, Shah RK (2023). Forest cover change detection using remote sensing and GIS in Dibru-Saikhowa National Park, Assam: a spatio-temporal study. Proc Natl Acad Sci India Sect B Biol Sci.

[29] Haq SM, Khoja AA, Lone FA, Waheed M, Bussmann RW, Casini R, Mahmoud EA, Elansary HO (2023). Keeping healthy in your skin—plants and fungi used by indigenous Himalayan communities to treat dermatological ailments. Plants.

[30] McDonald T, Gann GD, Jonson J, Dixon KW, Aronson J, Decleer K, Hallett J, Keenleyside K, Nelson C, Walder B, Wickwire L. International standards for the practice of ecological restoration–including principles and key concepts. Soc Ecolog Restorat. 2016.

[31] Haq SM, Hassan M, Bussmann RW, Calixto ES, Rahman IU, Sakhi S, Ijaz F, Hashem A, Al-Arjani ABF, Almutairi KF, Abd-Allah EF (2020). A cross-cultural analysis of plant resources among five ethnic groups in the Western Himalayan region of Jammu and Kashmir. Biology.

[32] Khoja AA, Haq SM, Majeed M, Hassan M, Waheed M, Yaqoob U, Bussmann RW, Alataway A, Dewidar AZ, Al-Yafrsi M, Elansary HO (2022). Diversity, Ecological and traditional knowledge of pteridophytes in the western Himalayas. Diversity.

[33] Haq SM, Waheed M, Khoja AA, Amjad MS, Bussmann RW, Ali K (2023). A cross-cultural study of high-altitude botanical resources among diverse ethnic groups in Kashmir Himalaya, India. J Ethnobiol Ethnomed.

[34] Phillips JM (1994). Farmer education and farmer efficiency: a meta-analysis. Econ Develop Cult Chan.

[35] Waheed M, Haq SM, Arshad F, Bussmann RW, Pieroni A, Mahmoud EA, Casini R, Yessoufou K, Elansary HO (2023). Traditional wild food plants gathered by ethnic groups living in semi-arid region of Punjab, Pakistan. Biology.

[36] Sorensen TA. A method of establishing groups of equal amplitude in plant sociology based on similarity of species content and its application to analyses of the vegetation on Danish commons. Kongelige Danske videnskabernes selskab.; Biologiske skrifter 1948; 5:1–34.

[37] Harsha R, Shuchi UAS (2020). An overview of *Dillenia indica* and their properties. T Phar Innovat J.

[38] Haq SM, Amjad MS, Waheed M, Bussmann RW, Proćków J (2022). The floristic quality assessment index as ecological health indicator for forest vegetation: a case study from Zabarwan Mountain Range. Himalayas Ecol Indic.

[39] Haq SM, Waheed M, Khoja AA, Amjad MS, Bussmann RW, Ali K, Jones DA (2023). Measuring forest health at stand level: A multi-indicator evaluation for use in adaptive management and policy. Ecol Indic.

[40] Lu Y, Ranjitkar S, Harrison RD, Xu J, Ou X, Ma X, He J (2017). Selection of native tree species for subtropical forest restoration in Southwest China. PLoS ONE.

[41] Elliott S, Navakitbumrung P, Kuarak C, Zangkum S, Anusarnsunthorn V, Blakesley D (2003). Selecting framework tree species for restoring seasonally dry tropical forests in northern Thailand based on field performance. Forest Ecol Manag.

[42] Krishnamurthy YL, Prakasha HM, Nanda A, Krishnappa M, Dattaraja HS, Suresh HS (2010). Vegetation structure and floristic composition of a tropical dry deciduous forest in Bhadra Wildlife Sanctuary, Karnataka, India. Trop Ecol.

[43] Sen A, Johri T, Bisht NS (2008). Analysis of the effects of anthropogenic interferences on tree species composition in the forests of Dadra and Nagar Haveli, India. Curr Sci.

[44] de Arruda HL, dos Santos JF, Albuquerque UP, Ramos MA (2019). Influence of socioeconomic factors on the knowledge and consumption of firewood in the Atlantic Forest of northeast Brazil. Econ Bot.

[45] Dkhar M, Tiwari BK (2020). Traditional ecological knowledge of tribal communities of Northeast India. Biodiv J Biolog Diver.

[46] Durbecq A, Jaunatre R, Buisson E, Cluchier A, Bischoff A (2020). Identifying reference communities in ecological restoration: the use of environmental conditions driving vegetation composition. Restorat Ecol.

[47] Newmaster AF, Berg KJ, Ragupathy S, Palanisamy M, Sambandan K, Newmaster SG (2011). Local knowledge and conservation of seagrasses in the Tamil Nadu State of India. J Ethnobiol Ethnomed.

[48] Das S (2017). Ecological restoration and livelihood: contribution of planted mangroves as nursery and habitat for artisanal and commercial fishery. World Dev.

[49] Zizka A, Thiombiano A, Dressler S, Nacoulma BM, Ouédraogo A, Ouédraogo I, Ouédraogo O, Zizka G, Hahn K, Schmidt M (2015). Traditional plant use in Burkina Faso (West Africa): a national-scale analysis with focus on traditional medicine. J Ethnobiol Ethnomed.

[50] Moitree T, Deb D, Deb S (2016). Utilization pattern of fuelwood plants by the Halam community of Tripura, Northeast India. Energy Sour Part A Recov Util Environ Eff.

[51] Mariscal A, Tigabu M, Savadogo P, Odén PC (2022). Regeneration status and role of traditional ecological knowledge for cloud forest ecosystem restoration in ecuador. Forests.

[52] Guariguata MR, Rheingans R, Montagnini F (1995). Early woody invasion under tree plantations in Costa Rica: implications for forest restoration. Restor Ecol.

[53] Holl KD, Loik ME, Lin EH, Samuels IA (2000). Tropical montane forest restoration in Costa Rica: overcoming barriers to dispersal and establishment. Restor Ecol.

[54] Dollo M, Samal PK, Sundriyal RCA, Kumar K (2009). Environmentally sustainable traditional natural resource management and conservation in Ziro Valley, Arunachal Himalaya. India J Am Sci.

[55] Hazarika TK, Pongener M (2018). Potential wild edible fruits of Nagaland, North-east India and its significance in the livelihood and nutritional security of rural, indigenous people. Genet Res Crop Evol.

[56] Jeevaa SRDN, Roytre CL, Mishra BP. Traditional agricultural practices in Meghalaya, Northeast India. (2006).

[57] Berkes F, Colding J, Folke C (2000). Rediscovery of traditional ecological knowledge as adaptive management. Ecolog App.

[58] Nongrum M, Syiem B (2022). How traditional agriculture contributes to the global narrative for sustainability: a case from a community in Northeast India. J Agric Food Syst Comm Develop.

[59] Arjun MS, Antony R, Ali AA, Abhirami C, Sreejith MM (2021). Diversity of Pteridophyte Flora in Rajamala, Eravikulam National Park, Kerala, India. A J Envir Ecol.

[60] Islebe GA (2003). Traditional ecological knowledge and use of vegetation in southeastern Mexico: a case study from Solferino. Quintana Roo Biodivers Conservat.

[61] Suneeta C, Nagesha N, Nataraja A, Kandpal K, Muttu V, Hegde S (2021). Ethnobotanical importance of pteridophytes of agumbe ghats. Indian Fern J.

[62] Haq SM, Calixto ES, Yaqoob U, Ahmed R, Mahmoud AH, Bussmann RW, Mohammed OB, Ahmad K, Abbasi AM (2020). Traditional usage of wild fauna among the local inhabitants of Ladakh, Trans-Himalayan Region. Animals.

[63] Subashree K, Dar JA, Karuppusamy S, Sundarapandian S (2021). Plant diversity, structure and regeneration potential in tropical forests of Western Ghats, India. Acta Ecolog Sin.

[64] Parrotta JA (1993). Secondary forest regeneration on degraded tropical lands. Restoration of tropical forest ecosystems.

[65] Bhattacharjee K, Boro A, Das AK, Dutta U, Sarma GC. Phytogeography of Chirang Reserve Forest under Manas Biosphere Reserve in Assam (India). 2014:374–380.

[66] Sharma N, Sharma A, Deka B, Sinha A (2020). Chronic extraction of forest resources is threatening a unique wildlife habitat of Upper Brahmaputra Valley, northeastern India. Current Sci.

[67] Sop TK, Oldeland J, Bognounou F, Schmiedel U, Thiombiano A (2012). Ethnobotanical knowledge and valuation of woody plants species: a comparative analysis of three ethnic groups from the sub-Sahel of Burkina Faso. Environ Develop Sustainabil.

[68] Rai SC (2007). Traditional ecological knowledge and community-based natural resource management in northeast India. J Mount Scien.

[69] Kiran KR, Miti R, Amit P (2009). Reclaiming degraded land in India through the cultivation of medicinal plants. Botan Res Int.

[70] Singh AP, Singh A, Dwived PK (2011). Conservation and restoration strategies for sustainable use of degraded terrestrial ecosystem of central India. Indian J Scient Res.

[71] Fulbright TE (1996). a theoretical basis for planning woody plant control to maintain species diversity. J Range Manag Arch.

[72] Fuentes-Gutiérrez E, Lindig-Cisneros R (2023). Biocultural, productive, and ecocentric restoration in La Mintzita Spring-fed Wetland, Michoacán. México Ecolog Restorat.

[73] Ganqa NM, Scogings PF, Scogings JG (2005). Diet selection and forage quality factors affecting woody plant selection by black rhinoceros in the Great Fish River Reserve, South Africa. Sou Afri J Wild Res.

[74] Sathya M (2017). A contemporary assessment of tree species in Sathyamangalam Tiger Reserve, Southern India. Proc Int Acad Ecol Environm Sci.

[75] Mathew, George. A study of wood boring beetles In the Kerala part of Nilgiri biosphere reserve. Kerala Forest Research Institute (KFRI) Research Report 260 (2004).

[76] Thakur AK, Yadav DK, Jhariya MK (2016). Feeding behaviour and pugmark analysis of elephants in Sarguja, Chhattisgarh. J App Nat Sci.

[77] Obura DO, DeClerck F, Verburg PH, Gupta J, Abrams JF, Bai X, Bunn S, Ebi KL, Gifford L, Gordon C, Jacobson L (2023). Achieving a nature-and people-positive future. One Earth.

[78] Baloch MA, Khan SUD, Ulucak ZŞ (2020). Poverty and vulnerability of environmental degradation in Sub-Saharan African countries: what causes what?. Struct Chang Econ Dyn.

[79] Elwell TL, López-Carr D, Gelcich S, Gaines SD (2020). The importance of cultural ecosystem services in natural resource-dependent communities: implications for management. Ecosys Servic.

[80] Tabuti JR, Ticktin T, Arinaitwe MZ, Muwanika VB (2009). Community attitudes and preferences towards woody species: implications for conservation in Nawaikoke. Uganda Oryx.

[81] Díaz S, Fargione J, Chapin FS, Tilman D (2006). Biodiversity loss threatens human well-being. PLoS Biol.

[82] Rahman M, Roy B, Chowdhury GM, Hasan A, Saimun M, Reza S (2022). Medicinal plant sources and traditional healthcare practices of forest-dependent communities in and around Chunati Wildlife Sanctuary in southeastern Bangladesh. Environ Sustain.

[83] FAO (2020). Restoring the earth—the next decade.

[84] Mbah M, Ajaps S, Molthan-Hill P (2021). A systematic review of the deployment of indigenous knowledge systems towards climate change adaptation in developing world contexts: Implications for climate change education. Sustainability.

[85] Tang R, Gavin MC (2019). A classification of threats to traditional ecological knowledge and conservation responses. Conservat Soci.

[86] Redvers N, Poelina A, Schultz C, Kobei DM, Githaiga C, Perdrisat M, Prince D, Blondin BS (2020). Indigenous natural and first law in planetary health. Challenges.

[87] Farooquee NA, Majila BS, Kala CP (2004). Indigenous knowledge systems and sustainable management of natural resources in a high-altitude society in Kumaun Himalaya. India J Hum Ecol.

[88] Loh J, Harmon D. Biocultural diversity: threatened species, endangered languages. WWF Netherlands, Zeist Amsterdam, Netherlands. 2014.

[89] Latulippe N, Klenk N (2002). Making room and moving over: knowledge co-production, Indigenous knowledge sovereignty and the politics of global environmental change decision-making. Curr Opi Environ Sustain.

